# Interleukin-17 Can Induce Osteoarthritis in Rabbit Knee Joints Similar to Hulth's Method

**DOI:** 10.1155/2017/2091325

**Published:** 2017-07-26

**Authors:** Zili Wang, Chenhuang Zheng, Yunbin Zhong, Jinshen He, Xu Cao, Hansong Xia, Hongliang Ba, Pei Li, Song Wu, Cheng Peng

**Affiliations:** ^1^Department of Orthopaedic Surgery, The Third Xiangya Hospital of Central South University, Changsha, Hunan 410013, China; ^2^Department of Plastic Surgery, The Third Xiangya Hospital of Central South University, Changsha, Hunan 410013, China

## Abstract

Interleukin-17 (IL-17) is closely related to osteoarthritis (OA), but animal studies that employ IL-17 to induce OA are currently lacking. Therefore, this study evaluated the effect of IL-17 in the rabbit knee joint. The right knees served as the control group. The left knees were divided randomly into 4 groups: a Hulth group and 3 IL-17 groups (1-ng, 10-ng, and 50-ng groups). OA was induced in the Hulth group using Hulth's method. The IL-17 groups were injected with 1, 10, or 50 ng of IL-17 as indicated. Specimens were collected at 72 h, 1 week, 3 weeks, 6 weeks, and 12 weeks after surgery or the last injection. Subsequently, the following experiments were conducted: X-ray analysis, histological evaluation, and polymerase chain reaction (PCR) analysis of the mRNA expression levels of cartilage degeneration-related markers. At 12 weeks, like the Hulth group, the 10-ng and 50-ng IL-17 groups displayed typical manifestations of OA. The X-ray results, histological scores, and mRNA expression levels showed statistically significant differences between the control group and the 10-ng and 50-ng IL-17 groups. In sum, injecting 10 ng of IL-17 into the rabbit knee joint can induce OA similar to OA induced by Hulth's method.

## 1. Introduction

Osteoarthritis (OA) is a joint disease that is characterized by the degeneration of articular cartilage. OA often affects the underlying bone and involves pathological changes in the synovium, joint capsule, ligaments, and other structures. OA occurs in an omnibearing and multilayered pattern and is related to varying degrees to ageing [1]. However, the pathogenesis of OA is not fully understood. To explore the pathogenesis of OA, scholars have constructed a variety of OA models. Current OA models mainly include spontaneous models and experimentally induced models. There are primarily 2 types of spontaneous models: naturally occurring models (e.g., the Dunkin Hartley guinea pig [2]) and genetically modified models (e.g., collagen type IX alpha 1 gene knockout mice [3]). The experimentally induced models are created mainly through intra-articular induction (e.g., traumatic surgery [4] or the injection of a chemical substance [5]) and extra-articular induction (e.g., joint immobilization [6]). At present, one of the classic and most administered methods of constructing an OA model is the Hulth method, which involves severing the ligaments surrounding the knee joint and transecting the meniscus [7]. The Hulth method has a high success rate in simulating posttraumatic OA. However, this method requires anaesthesia and a professional surgical procedure. In addition, the Hulth method may lead to postoperative joint instability, traumatic inflammation, and a number of other factors; for these reasons, this approach may not be conducive to investigating the roles of individual factors in the pathogenesis of OA.

In recent years, synovitis-mediated cartilage degeneration has become a hot topic in research. In trauma- and rheumatic disease-induced synovitis, the number of inflammatory cells and the levels of cytokines produced by the inflammatory cells are increased. Examples of these cytokines include interleukin-1*β* (IL-1*β*), tumour necrosis factor-alpha (TNF-a), IL-6, and IL-17. Such cytokines induce the production of matrix metalloproteinases (MMPs), disintegrins, and metalloproteinases with thrombospondin motifs (ADAMTS) and inducible nitric oxide synthase (iNOS) in the joints, ultimately leading to cartilage degradation [8]. Therefore, the relationships between the levels of cytokines such as IL-1*β*, TNF-a, and IL-17 and cartilage injury are often studied. Although IL-1*β* is the most recognized proinflammatory cytokine [9], it has been shown that IL-17 is similar to IL-1*β* in inducing the degradation of cartilage extracellular matrix [10]. In joints, IL-17 is mainly produced by T helper 17 cells (Th17), which differentiate from CD4^+^ T cells. IL-17 stimulates fibroblasts and epithelial cells to release IL-1*β*, TNF-a, IL-6, and IL-8; IL-17 [11] then synergizes with the above inflammatory factors to upregulate the expression of a number of factors that promote the degradation of the cartilage matrix, including MMPs, ADAMTS, and nitric oxide (NO) [12]. In addition, IL-17 inhibits the production of synthesis-promoting substances, such as proteoglycan and collagen type II [13]. In the basic research conducted by Koshy et al., adding IL-17 to the medium of in vitro cultured cartilage resulted in the loss of cartilage collagen type II and a reduction in the extracellular matrix [14]. Chen et al. applied IL-17 antagonists to mouse joints that showed signs of early OA and found that the production of a variety of inflammatory factors was inhibited. Therefore, it is believed that IL-17 antagonists are beneficial for patients with OA [15]. In a clinical study, Liu et al. found that the IL-17 content was significantly higher in the knee joints of OA patients than in normal knees and that IL-17 was closely related to the pain level and severity of OA [16]. Chen et al. found that IL-17 was elevated in the serum and synovial fluid of patients with OA and that it was positively correlated with the severity of OA on imaging analysis [17]. Although IL-17 has been found to be closely related to OA, animal studies that employ IL-17 to induce OA are currently lacking. Therefore, in the present study, we used IL-17 to induce OA. The present study provides an experimental basis for the future investigation of the role of IL-17 in the pathogenesis of OA.

## 2. Materials and Methods

### 2.1. Grouping of Experimental Animals and Construction of an OA Model

The Medical Animal Care & Ethical Committee of the Third Xiangya Hospital approved this research (approval number: LLSC (LA) 2015-030).

A total of 60 healthy New Zealand rabbits weighing approximately 2 kg each were provided by the Experimental Animal Center of the Third Xiangya Hospital of Central South University. The right knees of the rabbits served as normal controls (control group, *n* = 60). The left knees of the rabbits were randomly divided into 4 experimental groups (*n* = 15 in each group): the Hulth group, the 1-ng IL-17 group (1-ng group), the 10-ng IL-17 group (10-ng group), and the 50-ng IL-17 group (50-ng group). The Hulth group was treated according to the modified Hulth method described by Rogart et al. [7]. Briefly, the rabbits were anaesthetized by injection of 30 mg/kg sodium pentobarbital (Sigma-Aldrich, Saint Louis, MO, USA) into the marginal ear vein and then placed in a supine position. The skin over the left knee joint was prepared and disinfected. After administering anaesthesia, an anteromedial incision was made to expose the articular cavity of the left knee. Subsequently, the anterior and posterior cruciate ligaments were transected, and the medial meniscus was excised. A haemostatic procedure was performed, and the surgical wound was closed layer by layer. In the 3 groups that received injections of IL-17 (Sigma-Aldrich), 1 ml of IL-17 solution was injected into the articular cavity of the left knee (the concentrations of IL-17 were 1-ng/ml, 10-ng/ml, and 50-ng/ml in normal saline). Each group was given 1 injection every 2 days, and a total of 3 injections were administered to each group. After the surgery, none of the wounded limbs were immobilized. The animals were placed in standard animal rearing cages and were allowed to move about and feed freely.

### 2.2. Radiological Evaluation

Specimens were collected at 72 h, 1 week, 3 weeks, 6 weeks, and 12 weeks after the surgery or after the last injection. The knee joints were subjected to anteroposterior and lateral X-ray examination (Hitachi Limited, DHF-155HII, Tokyo, Japan). The parameters were set as follows: 42 kV, 250 mA, 32 ms, and 80 cm film-focus distance. The results were evaluated using the Kellgren-Lawrence (K-L) grading scale [18]. The K-L scoring criteria use a scale from 0 to 4 and is defined as follows: 0 = normal; 1 = questionable narrowing of joint space and possible osteophytic lipping; 2 = definite presence of osteophytes, definite narrowing of joint space; 3 = moderate multiple osteophytes, definite narrowing of joint space, some sclerosis, and possible deformity of the bone contour; 4 = large osteophytes, marked narrowing of joint space, severe sclerosis, and clear deformity of the bone contour.

### 2.3. Histological Examination

The femoral condyle, tibial plateau, and patellar cartilage were divided into 2 equal parts. One part was fixed in 4% paraformaldehyde (Leagene Bio., Beijing, China) for 3 days and decalcified in 10% Na_2_EDTA (Sigma-Aldrich) for 30 days. Subsequently, paraffin sections (thickness: 5 *μ*m) were prepared and subjected to conventional safranin O staining (Solarbio, Beijing, China) and haematoxylin and eosin (H&E) staining (Solarbio). In addition, the synovium of the infrapatellar fat pad was collected and subjected to conventional H&E staining. The histological results of the cartilage and synovium were analysed using ImageJ software (National Institutes of Health, Bethesda, MD, USA) and evaluated based on Mankin's scoring system [19] and on the histological scoring system for the synovial membrane [20]. Mankin's scoring system grades osteoarthritic cartilage on a scale from 0 (normal) to 14 points (severe OA) using the sum total score from these individually graded categories: structural compromise (0-6 points), loss of matrix staining (0-4), cellularity anomalies (0-3), and the violation of tidemark integrity (0-1). The synovial membrane histological scoring system consists of two grades, one for inflammation and one for proliferation. The two grades are combined and then divided by 2 for the official score. Inflammation was assessed from 0 to 4 based on the following criteria: 0 = no inflammatory cells; 1 = few, scattered cells; 2 = cells clearly present; 3 = multiple inflammatory cells organized in bands; 4 = massive presence of inflammatory cells. The synovial proliferation was also scored from 0 to 4 using the following criteria: 0 = none; 1 = small areas with synovial proliferation; 2 = large areas with synovial proliferation; 3 = invasion into the joint cavity by synovial proliferation; 4 = joint cavity completely occupied by synovial proliferation.

### 2.4. RNA Extraction and Reverse Transcription-Polymerase Chain Reaction (RT-PCR) Examination

Total RNA was extracted from the other portion of the cartilage samples using an RNeasy Mini kit (Qiagen, Dusseldorf, Germany). The expression levels of MMP-1, MMP-3, MMP-13, ADAMTS-4, ADAMTS-5, collagen type II alpha 1 chain (COL2A1), cartilage oligomeric matrix protein (COMP), collagen type I alpha 2 chain (COL1A2), and collagen type X alpha 1 chain (COL10A1) mRNA in the articular cartilage were determined by RT-PCR assays using a SYBR green kit (Sigma-Aldrich) according to the method described by Takahito [21] and Hyuck [22]. The PCR protocol involved heating at 50°C for 2 min and then 95°C for 10 min. Subsequently, the cycling conditions were optimized to 40 cycles of the following protocol: 95°C for 15 sec and 60°C for 60 sec. Cartilage from each time point was analysed 3 times, and the mean cycle threshold (Ct) value was obtained. Then, the average Ct values were normalized to the average Ct value of the housekeeping gene 18s. Relative expression levels were calculated using the formula 2^−△△Ct^. The sequences of the PCR primers are shown in Table 1.

### 2.5. Data Analysis

All data are expressed as the means ± standard deviation (*X* ± *S*). Statistical analysis was performed using the SPSS13.0 software package (IBM, Armonk, NY, USA). The paired* t*-test was employed.* P* values less than 0.05 were considered statistically significant.

## 3. **Results**

### 3.1. Gross Observations

**Figure 1:**
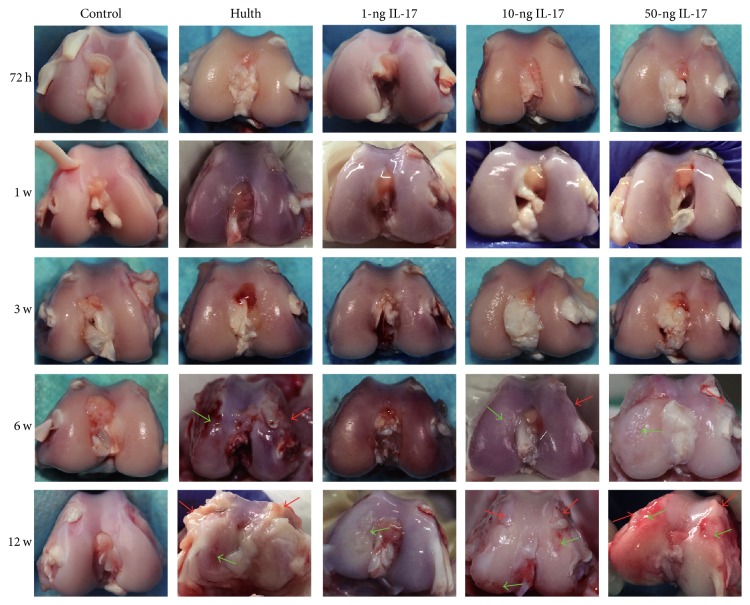
At all 5 time points, the articular cartilage in the control group displayed a glossy and uninterrupted surface, with no osteophyte formation. At 72 h and 1 week, similar to the control group, the 4 experimental groups exhibited no signs of cartilage degeneration. At 3 weeks, the Hulth group and the 10-ng and 50-ng groups exhibited a dim and rough surface, whereas the cartilage in the 1-ng group exhibited reduced lustre and transparency. At 6 weeks and 12 weeks after operation, the femoral condyles of the Hulth group, the 10-ng IL-17 group, and the 50-ng IL-17 group showed characteristics of osteoarthritis, while only small-scale cartilage injuries were seen in the 1-ng group. Red arrows indicate osteophytes, and green arrows indicate cartilage injury.

A glossy and uninterrupted cartilage surface was seen in the control group at all 5 time points. Similar to the control group, all 4 experimental groups displayed no signs of cartilage degeneration or injury at 72 h or at 1 week. At 3 weeks, the cartilage in the Hulth group, the 10-ng group, and the 50-ng group started to display a dim surface lustre, whereas the cartilage in the 1-ng group exhibited reduced lustre and transparency. At 6 weeks, the Hulth group, the 10-ng group, and the 50-ng group all exhibited cartilage defects and osteophyte formation, whereas only 2 specimens in the 1-ng group showed cartilage surface roughening to some extent. At 12 weeks, the Hulth group, the 10-ng group, and the 50-ng group all exhibited apparent cartilage defects and osteophyte formation. However, the number of osteophytes and the severity of joint deformity were slightly reduced in the 10-ng and 50-ng groups compared to the Hulth group. In contrast, only 1 specimen in the 1-ng group showed small-scale cartilage injuries, and no obvious osteophyte formation was observed (Figure 1).

### 3.2. X-Ray Examination

**Figure 2:**
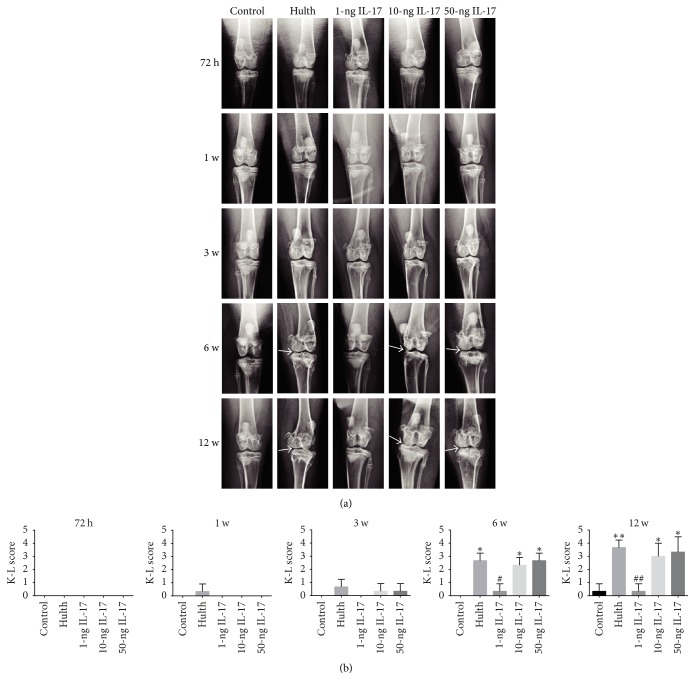
(a) Radiological evaluation shows that the control group exhibited normal knee joint signs at all 5 time points. Similar to the control group, the 4 experimental groups displayed no or mild signs of degeneration at 72 h, 1 week, and 3 weeks. At 6 weeks and 12 weeks, severe radiological signs of osteoarthritis were observed in the Hulth group, the 10-ng group, and the 50-ng group, while only a slight narrowing of the joint space and little osteophyte formation were seen in the 1-ng group. Arrows indicate narrowing of the joint space and osteophyte formation. (b) Kellgren-Lawrence scores (K-L score) for the severity of osteoarthritic lesions. ^*∗*^Compared to the control group, *P* < 0.05, ^*∗∗*^*P* < 0.01. ^#^Comparing the 3 IL-17 groups to the Hulth group, <0.05, ^##^*P* < 0.01.

At 72 h, 1 week, and 3 weeks, all 4 experimental groups appeared very similar to the control group. At 6 weeks, the 10-ng and 50-ng groups exhibited similarities to the Hulth group. X-ray examination revealed narrowing of the medial joint space and a low level of osteophyte formation accompanied by sclerosis and mild deformity of the subchondral bone. In contrast, the 1-ng group shared many similarities with the control group. The 1-ng group had a virtually normal joint space and showed no signs of osteophyte formation, subchondral bone sclerosis, or joint deformity. At 12 weeks, X-ray examination of the Hulth group revealed a significantly narrowed joint space and a high level of osteophyte formation accompanied by sclerosis and significant deformity of the subchondral bone. At that time, the 10-ng and 50-ng groups displayed some similarities to the Hulth group; however, osteophyte formation and joint deformity were relatively mild in these 2 groups. In contrast, the 1-ng group only exhibited mild joint space narrowing (Figure 2(a)).

The X-ray results were scored according to the K-L system. The K-L scores of all experimental groups increased gradually over time. At 3 weeks, there were no significant differences in K-L scores among the groups. At 6 and 12 weeks, the K-L score of the control group differed significantly from the scores of the Hulth group, the 10-ng group, and the 50-ng group (*P* < 0.05). However, no significant differences were found when the score of the Hulth group was compared with the scores of the 10-ng and the 50-ng groups. There was no significant difference in the K-L scores of the 1-ng group and the control group at these time points (*P* > 0.05), whereas there was a significant difference in the K-L scores of the 1-ng group and the Hulth group (*P* < 0.05) (Figure 2(b)).

### 3.3. Histomorphological Examination

#### 3.3.1. Histological Features of Cartilage

**Figure 3:**
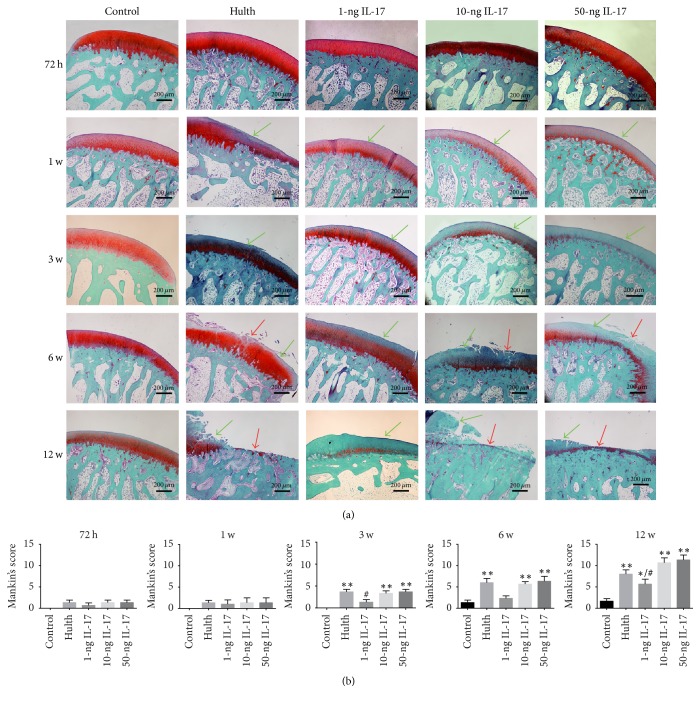
(a) The safranin O staining of cartilage shows that the control group exhibited consistent matrix staining, an integrated surface, and normal cellularity at all 5 time points. Similar to the control group, the 4 experimental groups exhibited no sign of degradation at 72 h. At 1 week, mildly reduced safranin O staining of the surface layer of the articular cartilage was seen in all groups except the control group, but cartilage injury or cellularity anomaly was not observed. At 3 weeks, 3 groups (the Hulth group, the 10-ng group, and the 50-ng group) exhibited significantly reduced safranin O staining at the surface of the articular cartilage, while the 1-ng group displayed mildly reduced safranin O staining. At 6 weeks and 12 weeks, the 3 groups showed histological signs of severe osteoarthritis compared with the control group, while the 1-ng group displayed obviously reduced safranin O staining but only mild cartilage injury. Green arrows indicate decreased staining, and red arrows indicate cartilage defects. (b) Mankin's score of cartilage. ^*∗*^Compared to the control group, *P* < 0.05, ^*∗∗*^*P* < 0.01. ^#^Comparing the 3 IL-17 groups to the Hulth group, *P* < 0.05, ^##^*P* < 0.01.

At 72 h, the chondrocytes displayed normal morphology and were arranged in an orderly fashion. In addition, a uniform distribution of safranin O staining was observed, and the tide line appeared intact. No significant differences existed among the groups. At 1 week, mildly reduced safranin O staining of the surface layer of the articular cartilage was observed in all groups except the control group. At 3 weeks, the Hulth group, the 10-ng group, and the 50-ng group all exhibited significantly reduced safranin O staining at the surface of the articular cartilage. In addition, the surface of the cartilage appeared uneven, and the subchondral bone displayed an increased number of lacunae. At 6 weeks, cracking of the cartilage layer, a decrease in the number of chondrocytes, and clustering of local diffuse hyperplasia were observed in the Hulth group, the 10-ng group, and the 50-ng group. At that time point, the cracks had propagated into the transitional layer or the calcified layer of the cartilage, and the tide line had partially disappeared; the intensity of safranin O staining was further reduced in the 1-ng group. At 12 weeks, partial or full-thickness cartilage injuries were detected in the Hulth group, the 10-ng group, and the 50-ng group. In addition, the number of chondrocytes was markedly reduced, safranin O staining was reduced or absent, and subchondral bone remodelling was complete. The above findings represent the typical manifestations of late-stage OA. Further reduction in safranin O staining and roughening of the cartilage surface was observed in the 1-ng group (Figure 3(a)).

Cartilage was evaluated using Mankin's scoring system. No significant differences among the groups were found at 72 h or at 1 week. Significant differences did exist between the control group and the 3 experimental groups (the Hulth group, the 10-ng group, and the 50-ng group) at 3 weeks, 6 weeks, and 12 weeks (*P* < 0.01). However, no significant differences were found when the Hulth group was compared with the 10-ng group or the 50-ng group. Although there was a significant difference between the 1-ng group and the control group at 12 weeks (*P* < 0.05), a significant difference between the 1-ng group and the Hulth group (*P* < 0.05) was also detected at 12 weeks (Figure 3(b)).

#### 3.3.2. Histological Features of the Synovium

**Figure 4:**
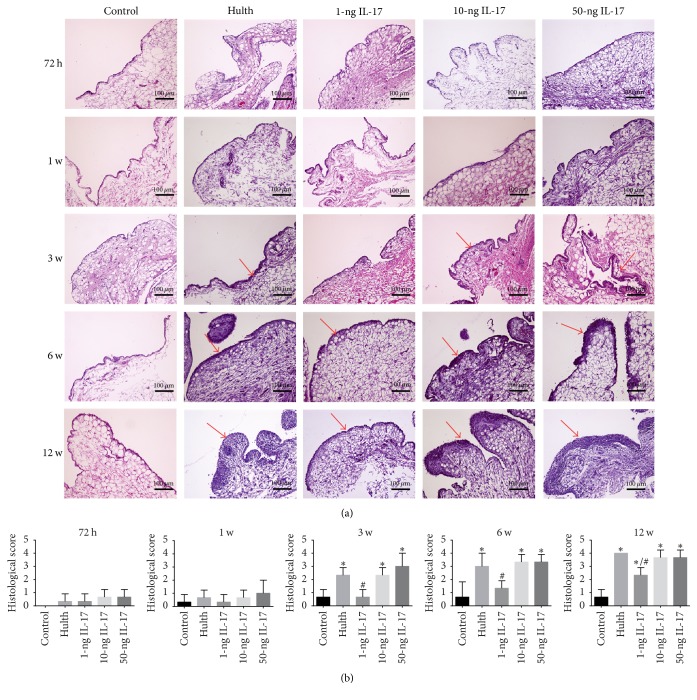
(a) H&E staining of the synovium shows that the control group displayed appropriate cell distribution and normal thickness at all 5 time points. At 72 h and at 1 week, the 4 experimental groups showed synovial histology results similar to the control group. At 3 weeks and 6 weeks, 3 groups (the Hulth group, the 10-ng group, and the 50-ng group) displayed significantly increased synovial thickness and cell number, while mild synovial hyperplasia was observed in the 1-ng group. At 12 weeks, the thickness of the synovium and the number of cells in the 3 abovementioned groups were further increased; meanwhile, the 1-ng group also exhibited evident synovial hyperplasia and inflammatory cell infiltration. Red arrows indicate synovial hyperplasia and inflammatory cell infiltration. (b) Histological scores of synovium. ^*∗*^Compared to the control group, *P* < 0.05, ^*∗∗*^*P* < 0.01. ^#^Comparing the 3 IL-17 groups to the Hulth group, *P* < 0.05, ^##^*P* < 0.01.

At 72 h and at 1 week, the 1-ng, 10-ng, 50-ng, and Hulth groups did not differ significantly from the control group in synovial thickness or cell number. At 3 weeks and 6 weeks, the synovial thickness and cell number were significantly increased in the Hulth group, the 10-ng group, and the 50-ng group compared to the control group. The 1-ng group displayed mild synovial hyperplasia. At 12 weeks, the thickness of the synovium and the number of cells in the Hulth group, the 10-ng group, and the 50-ng group were further increased and became significantly higher than in the control group. The 1-ng group also exhibited evident synovial hyperplasia and inflammatory cell infiltration. However, significant differences existed between the 1-ng group and the control group as well as between the 1-ng group and the Hulth group. Therefore, it was believed that the histological scores of the synovium in the 1-ng group would not reach the level of OA (Figure 4(a)).

The histological scores of the synovium revealed that there were no significant differences among the groups at 72 h or at 1 week. Significant differences were detected at 3 weeks, 6 weeks, and 12 weeks when comparing the control group with the Hulth group, the 10-ng group, and the 50-ng group (*P* < 0.05). In contrast, the histological scores of the 10-ng group and the 50-ng group were not significantly different from scores of the Hulth group at 3 weeks, 6 weeks, or 12 weeks. At 12 weeks, statistically significant differences existed between the 1-ng group and the control group (*P* < 0.05) as well as between the 1-ng group and the Hulth group (*P* < 0.05) (Figure 4(b)).

### 3.4. PCR Analysis of the Expression of MMP-1, MMP-3, MMP-13, ADAMTS-4, and ADAMTS-5 in Articular Cartilage

**Figure 5:**
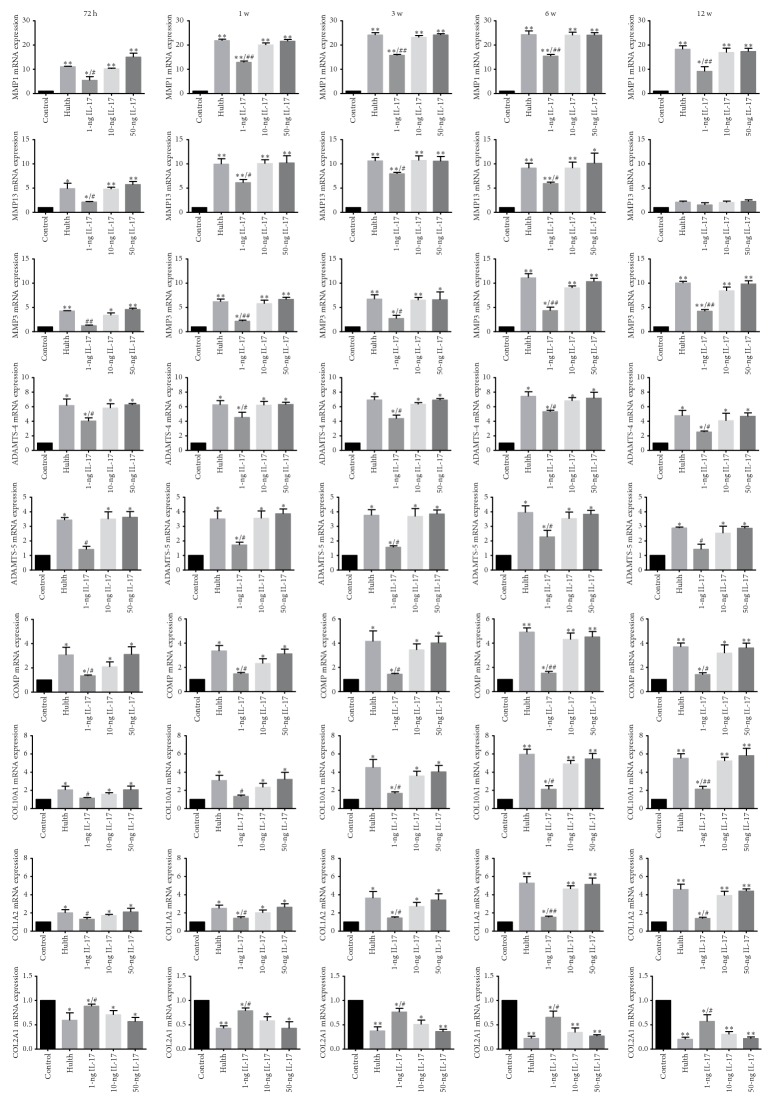
Graphs showing the RNA expression levels of MMP-1, MMP-3, MMP-13, ADAMTS-4, ADAMTS-5, COMP, COL10A1, COL1A2, and COL2A1 in articular cartilage. Compared to the control group, the expression of MMP-1, MMP-3, MMP-13, ADAMTS-4, ADAMTS-5, COMP, COL10A1, and COL1A2 was significantly increased at 72 h, 1 week, 3 weeks, and 6 weeks in the Hulth group and in the 3 groups injected with different concentrations of IL-17. At 12 weeks, MMP-13 expression was markedly reduced in all the experimental groups, whereas MMP-1, MMP-3, ADAMTS-4, ADAMTS-5, COMP, COL10A1, and COL1A2 expression remained high. COL2A1 expression was significantly reduced at all 5 time points in the Hulth group and in the 3 groups injected with different concentrations of IL-17. There were no significant differences in MMP-1, MMP-3, MMP-13, ADAMTS-4, ADAMTS-5, COMP, COL10A1, COL1A2, or COL2A1 expression levels between the Hulth group and the 10-ng group or the 50-ng group. ^*∗*^Compared to the control group, *P* < 0.05, ^*∗∗*^*P* < 0.01. ^#^Comparing the 3 IL-17 groups to the Hulth group, *P* < 0.05, ^##^*P* < 0.01.

Compared with the control group, MMP-1, MMP-3, and MMP-13 expression levels were significantly increased at 72 h, 1 week, 3 weeks, and 6 weeks in the Hulth group and in the 3 groups injected with different concentrations of IL-17. At 12 weeks, MMP-13 expression was markedly reduced in all experimental groups, whereas MMP-1 and MMP-3 expression remained high (compared to the control group, *P* < 0.01). PCR analysis of the expression of ADAMTS-4, ADAMTS-5, and COMP generated similar results. Compared to the control group, ADAMTS-4, ADAMTS-5, and COMP expression levels were significantly increased in the Hulth group and in the 3 groups injected with different concentrations of IL-17 (*P* < 0.05). The PCR analysis of the expression of COL10A1 and COL1A2 generated similar results. COL10A1 and COL1A2 expression levels were significantly increased (especially at 6 weeks and 12 weeks) in the Hulth group and in the 3 groups injected with different concentrations of IL-17 (compared to the control group, *P* < 0.05 at 72 h, 1 week, and 3 weeks, *P* < 0.01 at 6 weeks and 12 weeks). COL2A1 expression levels were significantly reduced in the Hulth group and in the 3 groups injected with different concentrations of IL-17 (compared to the control group, *P* < 0.05). No significant differences in MMP-1, MMP-3, MMP-13, ADAMTS-4, ADAMTS-5, COMP, COL10A1, COL1A2, or COL2A1 expression levels between the Hulth group and the 10-ng group or the 50-ng group were detected at any of the time points examined (*P* > 0.05). Although significant differences were detected between the 1-ng group and the control group at some time points, differences also existed between the 1-ng group and the Hulth group (Figure 5).

## 4. Discussion

In recent years, the relationship between inflammatory cytokines and OA has become an important research topic. IL-17 is one of the most important inflammatory cytokines and has been found to be closely related to OA. However, animal studies that employ IL-17 to induce OA and include a long-term quantitative analysis of the expression levels of IL-17-induced cartilage degeneration-related markers are currently lacking. Therefore, IL-17 was selected as an OA-inducing factor in the present study. Three experimental groups receiving different concentrations of IL-17 were set up and compared with a control group (normal group) and with a Hulth group. Using this strategy, we not only demonstrated that IL-17 is capable of inducing OA in vivo but also successfully determined the appropriate dose of IL-17 for this purpose. Moreover, the expression levels of cartilage degeneration-related markers induced by IL-17 in cartilage were examined at multiple time points.

OA is generally confirmed mainly through gross observation, histomorphological examination, and imaging examination. The main gross observation features of advanced OA are cartilage defects and osteophyte and joint deformities. The typical histological features are partial or full-thickness cartilage injuries, decreased numbers of chondrocytes, reduced saffron staining, and a thickened synovial membrane [22, 23]. In the present study, after the injection of IL-17 into rabbit knee joints in the 10-ng and 50-ng groups, typical gross and morphological features of late-stage OA were detected at 12 weeks. IL-17 induces synovitis through a number of mechanisms. For example, IL-17 can induce proinflammatory factors such as IL-1*β*, TNF-a, and IL-6, which jointly promote synovial hyperplasia. In addition, IL-17 stimulates synovial fibroblasts to produce vascular endothelial growth factor (VEGF), thereby promoting angiogenesis [24]. Some OA models have been subjected to imaging examination. Singh et al. established a rabbit model of OA through anterior cruciate ligament transection. X-ray examination showed that typical features of OA developed 16 weeks after the procedure, including apparent osteophyte formation, joint space narrowing, and joint deformity [25]. The present study also showed that the 10-ng and 50-ng groups displayed imaging features similar to the features observed in the Hulth group at 6 weeks and 12 weeks. The results of gross examination and X-ray examination showed that the 3 IL-17-injected groups displayed a slightly lower degree of osteophyte formation and joint deformity than did the Hulth group. This phenomenon occurs mainly because the Hulth method seriously impairs joint stability, unlike intra-articular injection. Joint instability is a main reason for osteophyte formation and joint deformity [26].

IL-17 acts on cartilage and enhances its catabolism. MMP-1, MMP-3, MMP-13, ADAMTS-4, and ADAMTS-5 are the most relevant enzymes in OA. MMP-1 and MMP-13 are collagenases of the MMP family. One basic study found that MMP-1 expression began to increase during the acute inflammatory phase (1 week after treatment) and was maintained at a high level. In contrast, MMP-13 expression reached a high level in the early and mid-stages of OA and then declined significantly during the late stage of OA [27, 28]. A clinical study reported that the expression of MMP13 was significantly increased in the cartilage of patients with grade I OA, whereas the MMP13 expression level was fairly low in the cartilage of patients with grade IV OA [29]. In the present study, analysis of MMP-1 and MMP-13 expression in the Hulth group, the 10-ng IL-17 group, and the 50-ng IL-17 group yielded similar results. MMP-3 is an interstitial lytic enzyme of the MMP family. Chang et al. established an animal model of posttraumatic OA and examined a large number of OA-related factors. It was found that MMP-3 expression in the cartilage began to increase at 72 h, reached a peak at 6 weeks, and declined at 12 weeks [30]. The changes reported by Chang et al. were similar to the changes in MMP-3 expression observed in the present study. ADAMTS-4 and ADAMTS-5 are 2 typical types of aggrecanases that cause proteoglycan degradation. In the present study, the ADAMTS-4 and ADAMTS-5 expression levels in the Hulth group, the 10-ng IL-17 group, and the 50-ng IL-17 group increased to high values at earlier time points than the MMPs, possibly because a precondition of collagen type II degradation in OA is proteoglycan decomposition [31]. OA is a type of joint disease that involves articular cartilage lesion and subchondral bone remodelling. COMP and COL2A1 are 2 cartilage extracellular matrix degradation-related markers, and COL10A1 and COL1A2 are 2 chondrocyte hypertrophy-related markers. It has been reported that the expression levels of COMP [32], COL10A1 [33], and COL1A2 [34] are significantly increased, while the expression level of COL2A1 [35] is obviously reduced in the inflammatory lesions of joint cartilage in OA. In the present study, the expression levels of COMP, COL2A1, COL10A1, and COL1A2 in the Hulth group, the 10-ng IL-17 group, and the 50-ng IL-17 group yielded similar results. In summary, the expression levels of MMP-1, MMP-3, MMP-13, ADAMTS-4, ADAMTS-5, COMP, COL2A1, COL10A1, and COL1A2 in the Hulth group and the 10-ng and 50-ng IL-17 groups were similar, which indicates that the knee joint inflammation caused by full-dose IL-17 is similar to that caused by posttraumatic OA.

## 5. Conclusions

The present study demonstrates that the injection of 10 ng/ml IL-17 into the rabbit knee joint cavity (1 injection every 2 days for a total of 3 injections) can induce OA similar to Hulth's method. The expression levels of MMP-1, MMP-3, MMP-13, ADAMTS-4, ADAMTS-5, COMP, COL2A1, COL10A1, and COL1A2 in the cartilage were similar to the levels that occur in OA. Our findings provide an experimental basis for future investigations of the role of IL-17 in the pathogenesis of OA.

## Figures and Tables

**Table 1:** Primers for the target genes.

Gene	Primers (5′ to 3′)	Strand	Product size (bp)	Reference
18s	CGGACAGGATTGACAGATTGATAGC	+	118	X00640.1
	TGCCAGAGTCTCGTTCGTTATCG	−		
MMP-1	TGTTCAGTGGTGATGTTCAGTTAGC	+	161	M25663.1
	TATTTCTCCCCGAATTGTGGTTATAGC	−		
MMP-3	AATGGACAAAGGATACAACAGGAACC	+	162	M25664.1
	CATCATCTTGAGAAAGGCGGAACC	−		
MMP-13	TGAGATCATACTACCATCCTCTGAATCC	+	123	AF059201.1
	CAAGTTTGCCTGTCACCTCTAAGC	−		
ADAMTS-4	GCACTGACCTCTTCAAGAACTTCC	+	131	AF247707
	TGGTTCCAGCAACGTAGTAGTAGC	−		
ADAMTS-5	CTTCCACTAAGTAGTCCATGTAGATTGC	+	96	AF247708
	GGTCATTCCGATGTGGATTGC	−		
COMP	GACTTCCGGGCCTTCCAGAC	+	115	S83370.1
	GGTCGCTGTTCATGGTCTGC	−		
COL10A1	GGAGAGCCAGGGTTGCCAG	+	91	AF325902.1
	GTCCTCTCTCCCCTTGTTTTCC	−		
COL1A2	TAAGAGCTCCAAGGCCAAGA	+	152	XM_001161595.1
	TGTTCTGAGAGGCGTGATTG	−		
COL2A1	GGTGTGAGTCCAACGCCCCGCCC	+	95	NM_001195668.1
	GTTTGACACGGAGTAGCACCATC	−		

## References

[1] Saarakkala S., Julkunen P., Kiviranta P., Mäkitalo J., Jurvelin J. S., Korhonen R. K. (2010). Depth-wise progression of osteoarthritis in human articular cartilage: investigation of composition, structure and biomechanics. *Osteoarthritis and Cartilage*.

[2] Lampropoulou-Adamidou K., Lelovas P., Karadimas E. V. (2014). Useful animal models for the research of osteoarthritis. *European Journal of Orthopaedic Surgery and Traumatology*.

[3] Allen K. D., Griffin T. M., Rodriguiz R. M. (2009). Decreased physical function and increased pain sensitivity in mice deficient for type IX collagen. *Arthritis and Rheumatism*.

[4] Piskin A., Gulbabar M. Y., Tomak Y. (2007). Osteoarthritis models after anterior cruciate ligament resection and medial meniscectomy in rats: a histological and immunohistochemical study. *Saudi Medical Journal*.

[5] de Morais S. V., Czeczko N. G., Malafaia O. (2016). Osteoarthritis model induced by intra-articular monosodium iodoacetate in rats knee. *Acta Cirurgica Brasileira*.

[6] Zhou Q., Wei B., Liu S. (2015). Cartilage matrix changes in contralateral mobile knees in a rabbit model of osteoarthritis induced by immobilization. *BMC Musculoskeletal Disorders*.

[7] Rogart J. N., Barrach H.-J., Chichester C. O. (1999). Articular collagen degradation in the Hulth-Telhag model of osteoarthritis. *Osteoarthritis and Cartilage*.

[8] Kapoor M., Martel-Pelletier J., Lajeunesse D., Pelletier J., Fahmi H. (2011). Role of proinflammatory cytokines in the pathophysiology of osteoarthritis. *Nature Reviews Rheumatology*.

[9] Zhong Y., Huang Y., Santoso M. B., Wu L.-D. (2015). Sclareol exerts anti-osteoarthritic activities in interleukin-1*β*-induced rabbit chondrocytes and a rabbit osteoarthritis model. *International Journal of Clinical and Experimental Pathology*.

[10] Dudler J., Renggli-Zulliger N., Busso N., Lotz M., So A. (2000). Effect of interleukin 17 on proteoglycan degradation in murine knee joints. *Annals of the Rheumatic Diseases*.

[11] Magyari L., Varszegi D., Kovesdi E. (2014). Interleukins and interleukin receptors in rheumatoid arthritis: Research, diagnostics and clinical implications. *World Journal of Orthopaedics*.

[12] Pacquelet S., Presle N., Boileau C. (2002). Interleukin 17, a nitric oxide-producing cytokine with a peroxynitrite-independent inhibitory effect on proteoglycan synthesis. *Journal of Rheumatology*.

[13] Lubberts E., Joosten L. A. B., Van De Loo F. A. J., Van Den Bersselaar L. A. M., Van Den Berg W. B. (2000). Reduction of interleukin-17-induced inhibition of chondrocyte proteoglycan synthesis in intact murine articular cartilage by interleukin-4. *Arthritis and Rheumatism*.

[14] Koshy P. J., Henderson N., Logan C., Life P. F., Cawston T. E., Rowan A. D. (2002). Interleukin 17 induces cartilage collagen breakdown: novel synergistic effects in combination with proinflammatory cytokines. *Annals of the Rheumatic Diseases*.

[15] Chen L., Li D. Q., Zhong J. (2011). IL-17RA aptamer-mediated repression of IL-6 inhibits synovium inflammation in a murine model of osteoarthritis. *Osteoarthritis and Cartilage*.

[16] Liu Y., Peng H., Meng Z., Wei M. (2015). Correlation of IL-17 level in synovia and severity of knee osteoarthritis. *Medical Science Monitor*.

[17] Chen B., Deng Y., Tan Y., Qin J., Chen L.-B. (2014). Association between severity of knee osteoarthritis and serum and synovial fluid interleukin 17 concentrations. *The Journal of International Medical Research*.

[18] Kellgren J. H., Lawrence J. S. (1957). Radiological assessment of osteo-arthrosis. *Annals of the Rheumatic Diseases*.

[19] Mankin H. J., Dorfman H., Lippiello L., Zarins A. (1971). Biochemical and metabolic abnormalities in articular cartilage from osteo-arthritic human hips. *Journal of Bone and Joint Surgery*.

[20] Kristensen K. D., Stoustrup P., Küseler A. (2008). Quantitative histological changes of repeated antigen-induced arthritis in the temporomandibular joints of rabbits treated with intra-articular corticosteroid. *Journal of Oral Pathology and Medicine*.

[21] Yuasa T., Otani T., Koike T., Iwamoto M., Enomoto-Iwamoto M. (2008). Wnt/*β*-catenin signaling stimulates matrix catabolic genes and activity in articular chondrocytes: Its possible role in joint degeneration. *Laboratory Investigation*.

[22] Kwon H. J., Akimoto H., Ohmiya Y., Honma K., Yasuda K. (2008). Gene expression profile of rabbit cartilage by expressed sequence tag analysis. *Gene*.

[23] Huebner K. D., Shrive N. G., Frank C. B. (2013). New surgical model of post-traumatic osteoarthritis: Isolated intra-articular bone injury in the rabbit. *Journal of Orthopaedic Research*.

[24] Honorati M. C., Cattini L., Facchini A. (2007). VEGF production by osteoarthritic chondrocytes cultured in micromass and stimulated by IL-17 and TNF-*α*. *Connective Tissue Research*.

[25] Singh A., Goel S. C., Gupta K. K. (2014). The role of stem cells in osteoarthritis: an experimental study in rabbits. *Bone and Joint Research*.

[26] Grifka J., Ogilvie-Harris D. J.

[27] Green D. M., Noble P. C., Bocell J. R., Ahuero J. S., Poteet B. A., Birdsall H. H. (2006). Effect of early full weight-bearing after joint injury on inflammation and cartilage degradation. *Journal of Bone and Joint Surgery*.

[28] Fernandes J. C., Martel-Pelletier J., Lascau-Coman V., Moldovan F., Jovanovic D., Raynauld J. (1998). Collagenase-1 and collagenase-3 synthesis in normal and early experimental osteoarthritic canine cartilage: an immunohistochemical study. *The Journal of Rheumatology*.

[29] Barreto G., Soininen A., Ylinen P. (2015). Soluble biglycan: A potential mediator of cartilage degradation in osteoarthritis. *Arthritis Research and Therapy*.

[30] Chang J. C., Sebastian A., Murugesh D. K. (2016). Global molecular changes in a tibial compression induced ACL rupture model of post-traumatic osteoarthritis. *Journal of Orthopaedic Research*.

[31] Pratta M. A., Yao W., Decicco C. (2003). Aggrecan Protects Cartilage Collagen from Proteolytic Cleavage. *Journal of Biological Chemistry*.

[32] Helmark I. C., Petersen M. C. H., Christensen H. E., Kjaer M., Langberg H. (2012). Moderate loading of the human osteoarthritic knee joint leads to lowering of intraarticular cartilage oligomeric matrix protein. *Rheumatology International*.

[33] Gevers G., Dequeker J., Martens M., Van Audekercke R., Nyssen-Behets C., Dhem A. (1989). Biomechanical characteristics of iliac crest bone in elderly women according to osteoarthritis grade at the hand joints. *Journal of Rheumatology*.

[34] Couchourel D., Aubry I., Delalandre A. (2009). Altered mineralization of human osteoarthritic osteoblasts is attributable to abnormal type I collagen production. *Arthritis and Rheumatism*.

[35] Zhong L., Huang X., Karperien M., Post J. N. (2016). Correlation between gene expression and osteoarthritis progression in human. *International Journal of Molecular Sciences*.

